# Supplementary material for: Meroterpenoids from Gongolaria abies-marina against Kinetoplastids: In Vitro Activity and Programmed Cell Death Study
Source: Pharmaceuticals (Basel). 2023 Mar 23;16(4):476. doi: 10.3390/ph16040476 (PMC10146491; doi:10.3390/ph16040476)
Supplement: Supplementary file 1 [file pharmaceuticals-16-00476-s001.zip › pharmaceuticals-2288893-supplementary.pdf]

Supplementary Material

Plasmatic membrane permeability assay and chromatin condensation analysis

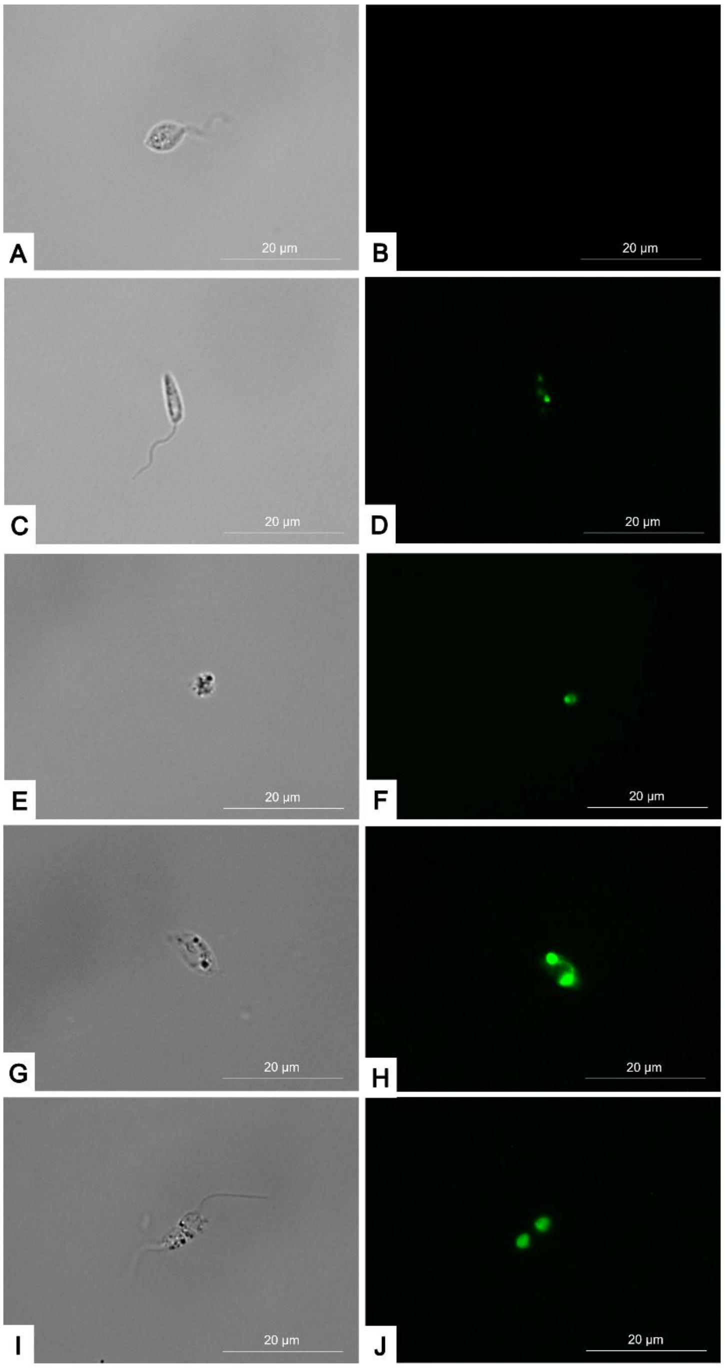

Figure S1**.** *L. amazonensis* promastigotes incubated with IC_{90} of cystomexicone B (C, D), gongolarone B (E, F), 6Z-1'-methoxyamentadione (G, H) and 1'-methoxyamentadione (I, J) during 24 h. Negative control of the parasites without any treatment (A, B). The 100X images were captured using an EVOS® FL Cell Imaging System. Scale-bar: 20 µm.

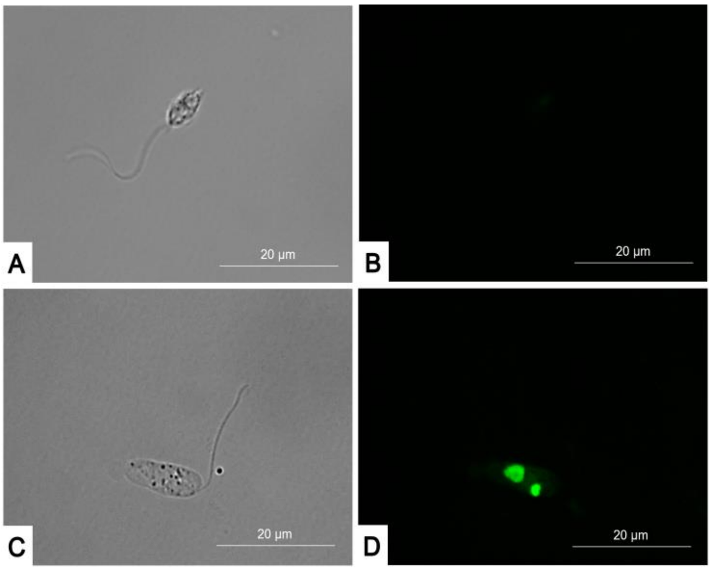

Figure S2. *L. donovani* promastigotes incubated with IC_{90} of cystomexicone B (C, D) during 24 h. Negative control of the parasites without any treatment (A, B). The 100X images were captured using an EVOS® FL Cell Imaging System. Scale-bar: 20 µm.

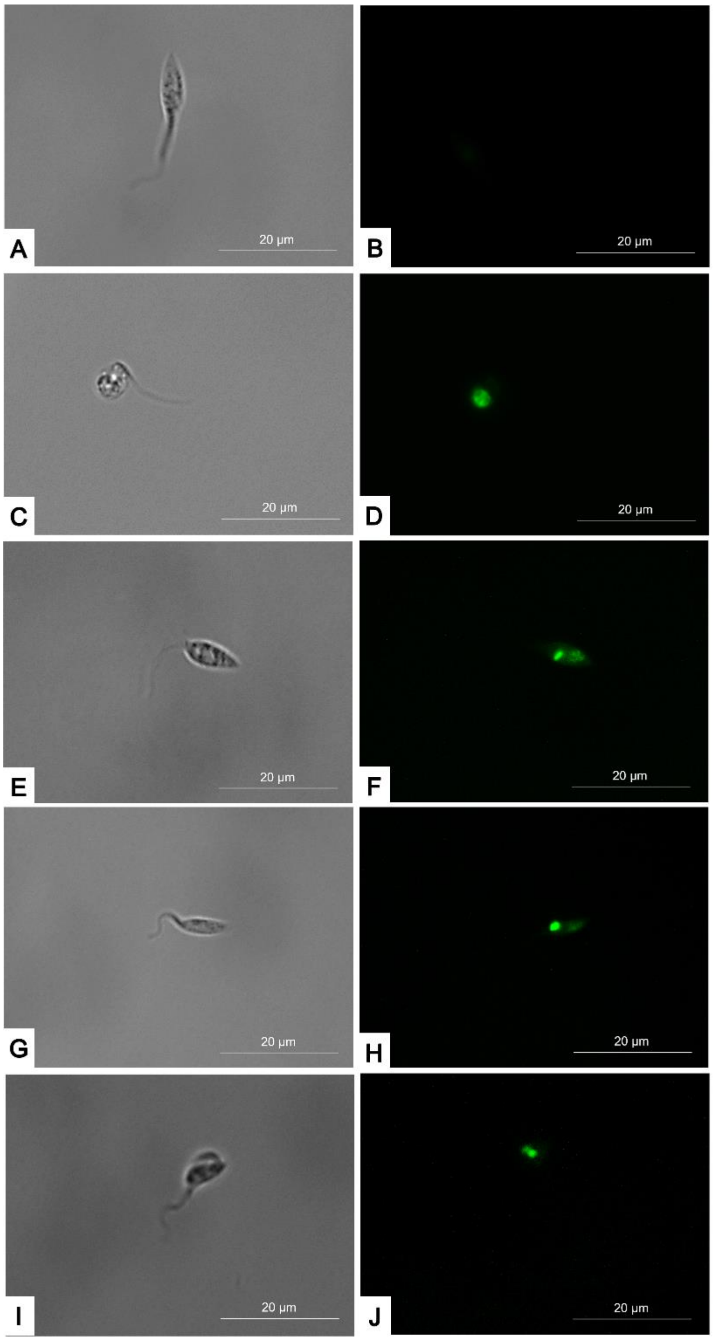

Figure S3. *T. cruzi* epimastigotes incubated with IC_{90} of cystomexicone B (C, D), gongolarone B (E, F), 6*Z*-1'-methoxyamentadione (G, H) and 1'-methoxyamentadione (I, J) during 24 h. Negative control of the parasite without any treatment (A, B). The 100X images were captured using an EVOS® FL Cell Imaging System. Scale-bar: 20 µm.

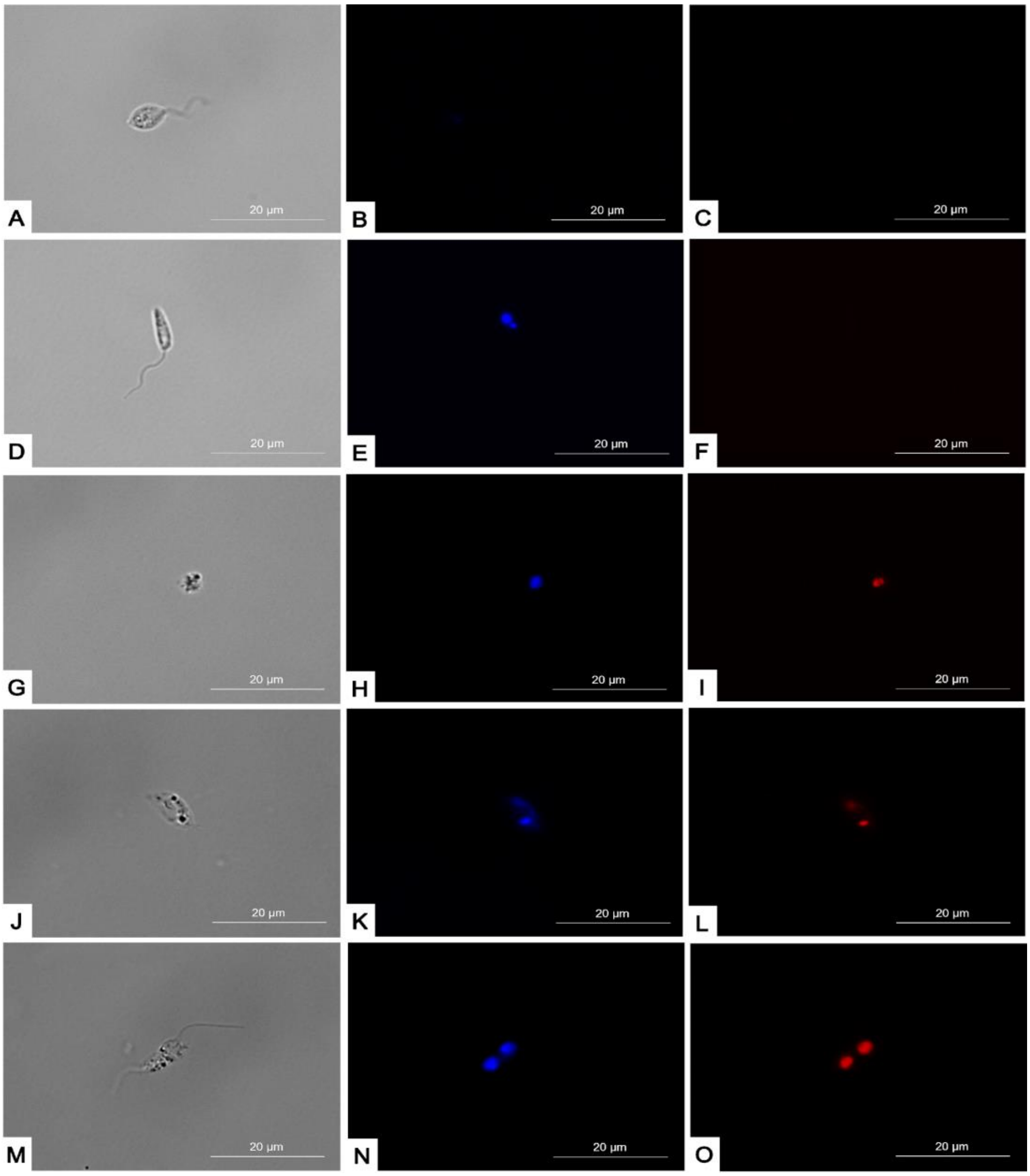

Figure S4. *L. amazonensis* promastigotes incubated with IC_{90} of cystomexicone B (D, E, F), gongolarone B (G, H, I), 6*Z*-1'-methoxyamentadione (J, K, L) and 1'-methoxyamentadione (M, N, O) during 24 h. Negative control of the parasites without any treatment (A, B, C). The 100X images were captured using an EVOS® FL Cell Imaging System. Scale-bar: 20 µm.

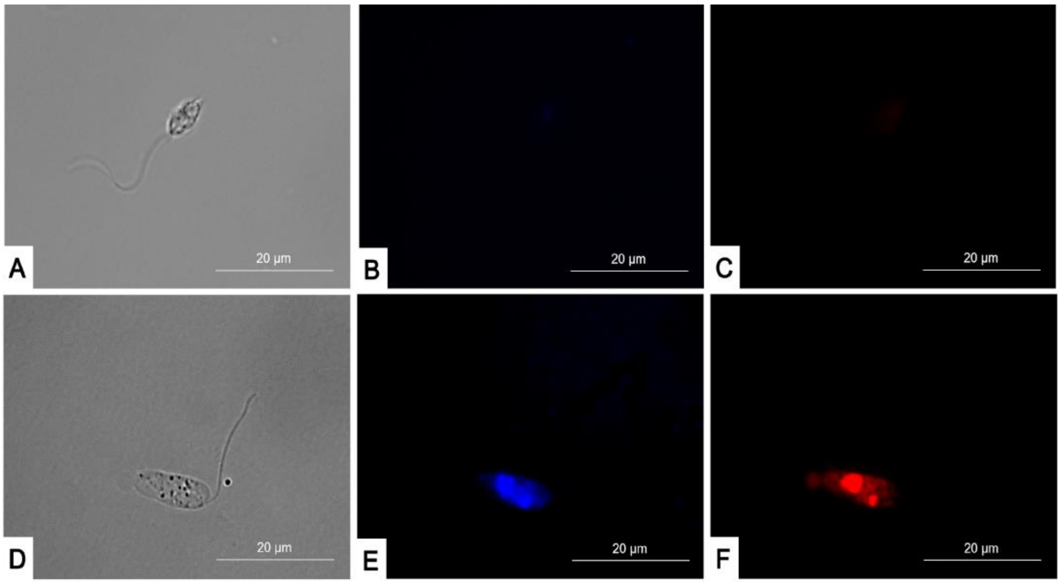

Figure S5. *L. donovani* promastigotes incubated with IC_{90} of cystomexicone B (D, E, F) during 24 h. Negative control of the parasites without any treatment (A, B, C). The 100X images were captured using an EVOS® FL Cell Imaging System. Scale-bar: 20 µm.

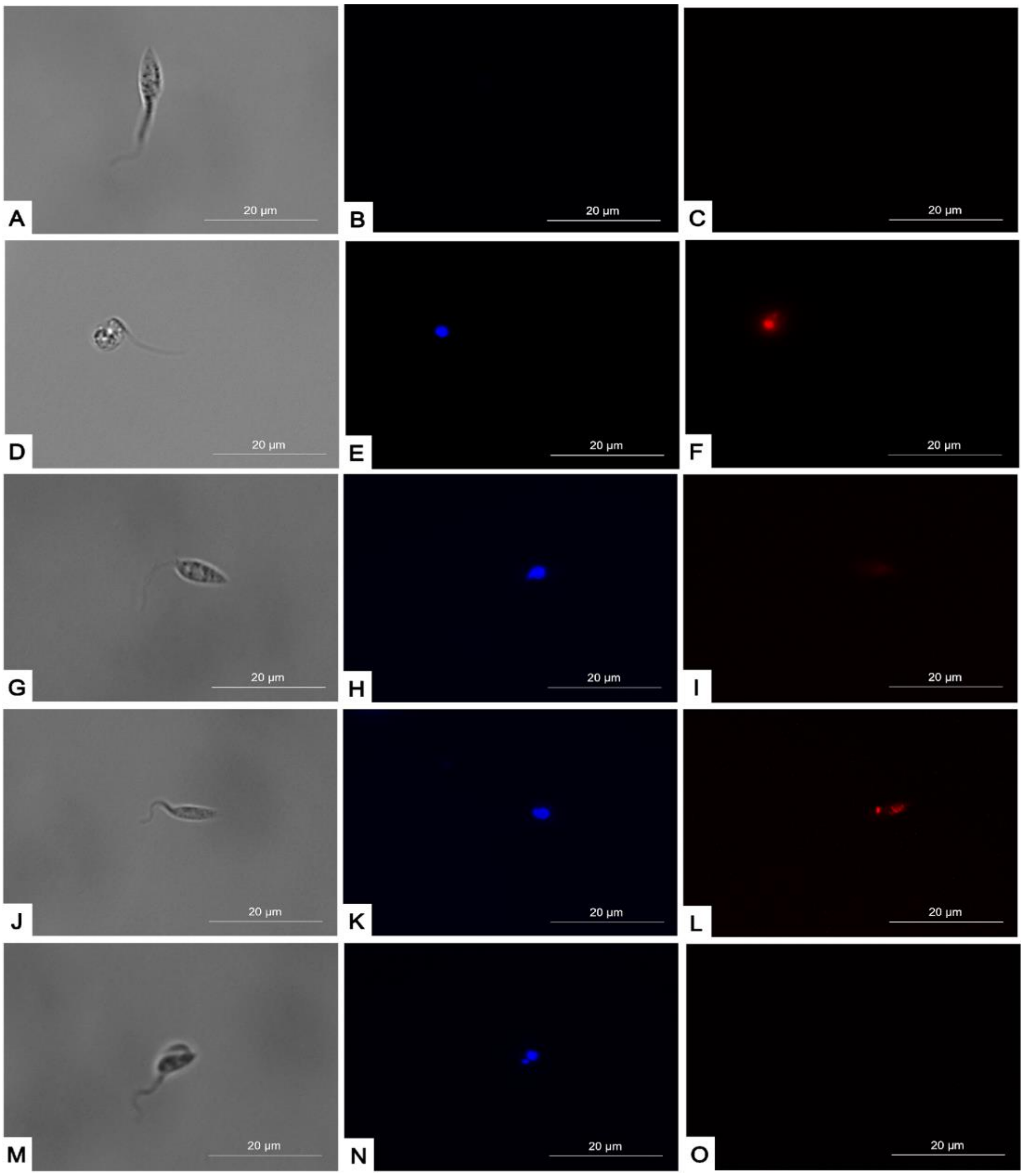

Figure S6. *T. cruzi* epimastigotes incubated with IC_{90} of cystomexicone B (D, E, F), gongolarone B (G, H, I), 6Z-1'-methoxyamentadione (J, K, L) and 1'-methoxyamentadione (M, N, O) during 24 h. Negative control of the parasite without any treatment (A, B, C). The 100X images were captured using an EVOS® FL Cell Imaging System. Scale-bar: 20 µm.

Analysis of reactive oxygen species

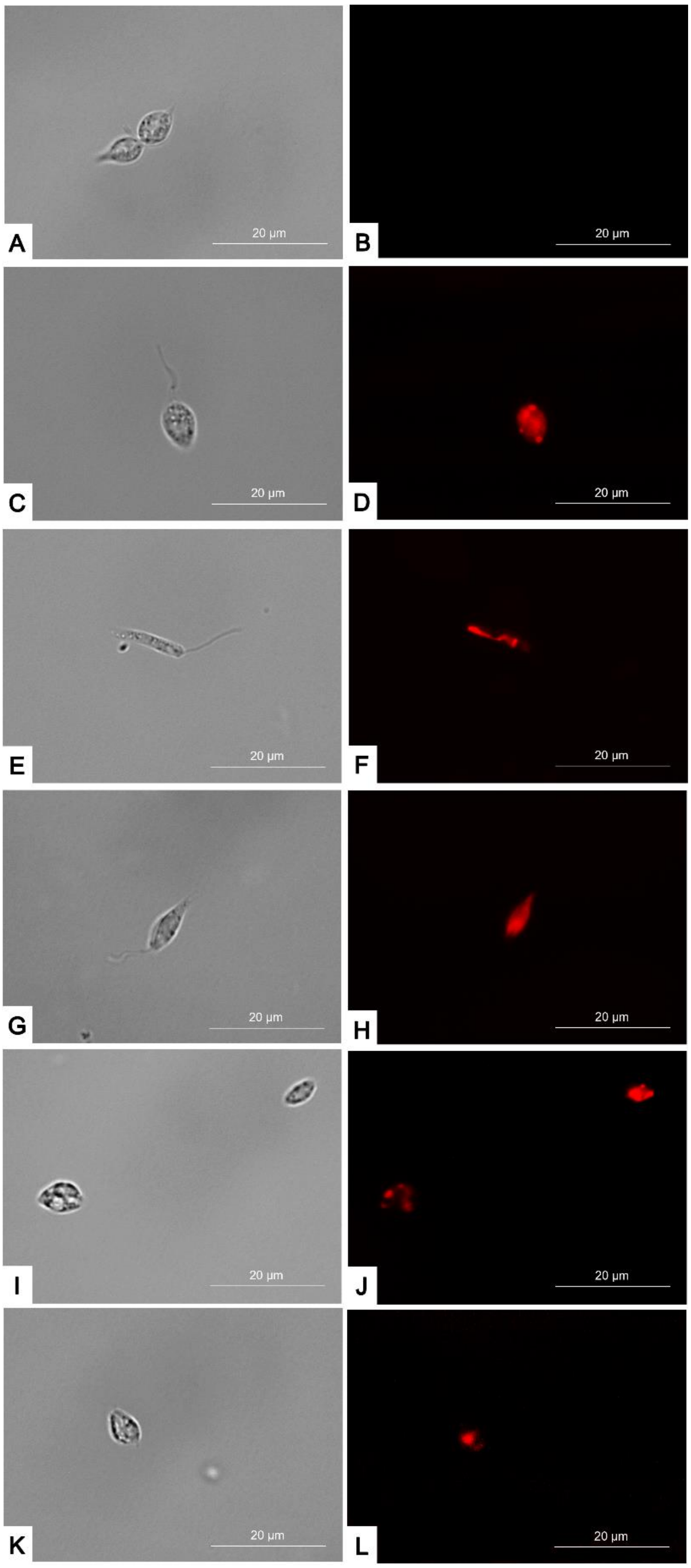

Figure S7. *L. amazonensis* promastigotes incubated with IC_{90} of cystomexicone B (E, F), gongolarone B (G, H), 6*Z*-1'-methoxyamentadione (I, J) and 1'-methoxyamentadione (K, L) during 24 h. Negative control of the parasite without any treatment (A, B) and the positive control with

H_2O_2 at 600 μM (C,D). The 100X images were captured using an EVOS® FL Cell Imaging System. Scale-bar: 20 μm.

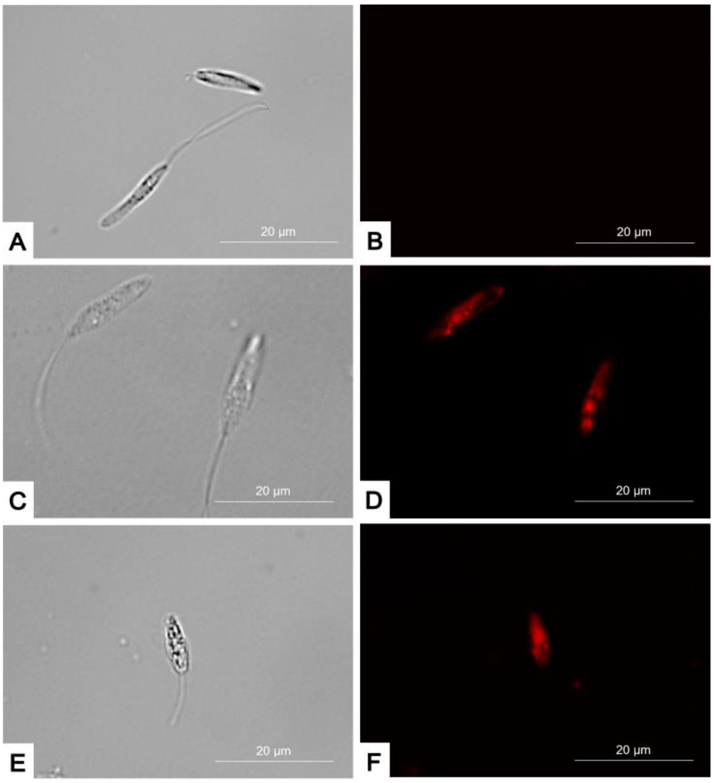

Figure S8. *L. donovani* promastigotes incubated with IC_{90} of cystomexicone B (E, F), during 24 h. Negative control of the parasite without any treatment (A, B) and the positive control with H_2O_2 at 600 μM (C, D). The 100X images were captured using an EVOS® FL Cell Imaging System. Scale-bar: 20 μm.

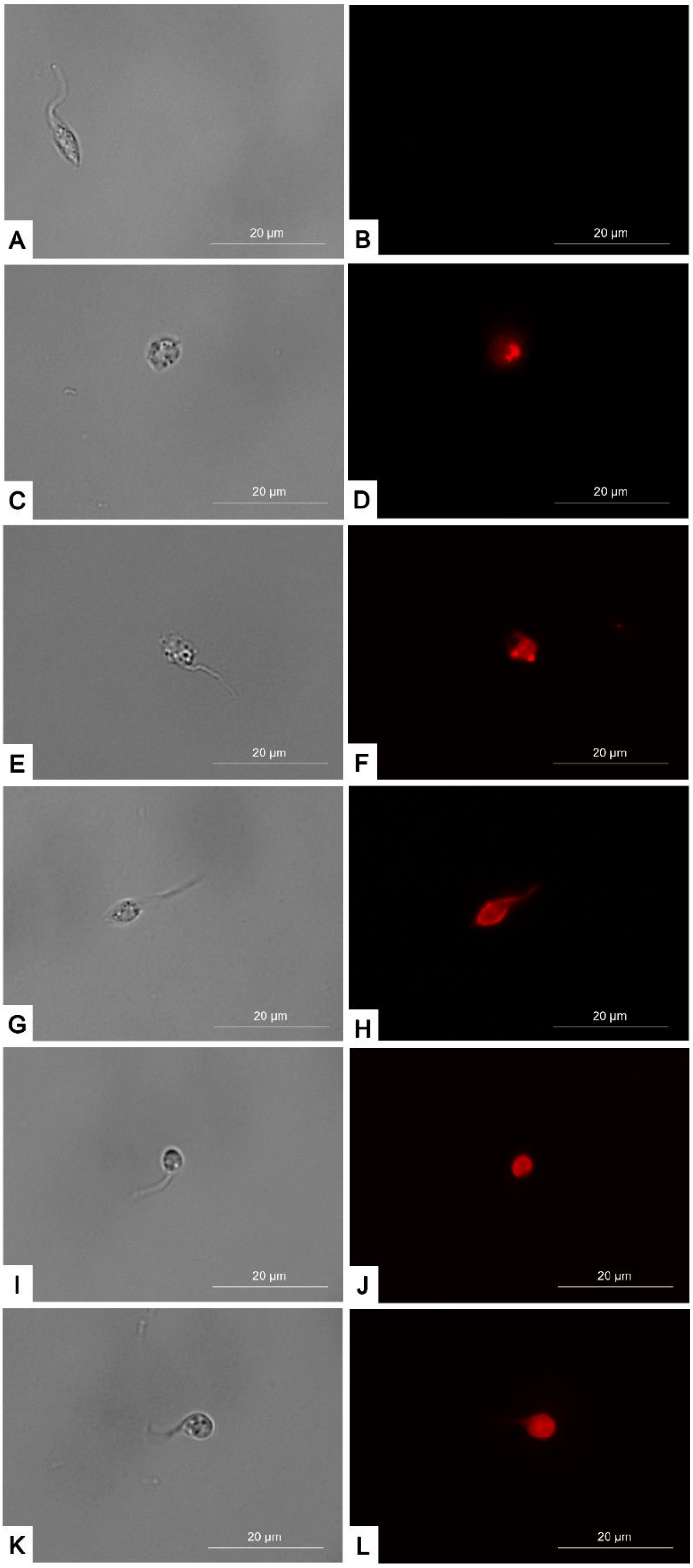

Figure S9. *T. cruzi* epimastigotes incubated with IC_{90} of cystomexicone B (E, F), gongolarone B (G, H), 6Z-1'-methoxyamentadione (I, J) and 1'-methoxyamentadione (K, L) during 24 h. Negative control of the parasite without any treatment (A, B) and the positive control with H_2O_2 at 600 µM (C, D). The 100X images were captured using an EVOS® FL Cell Imaging System. Scale-bar: 20 µm.

Immunofluorescence analysis of actin

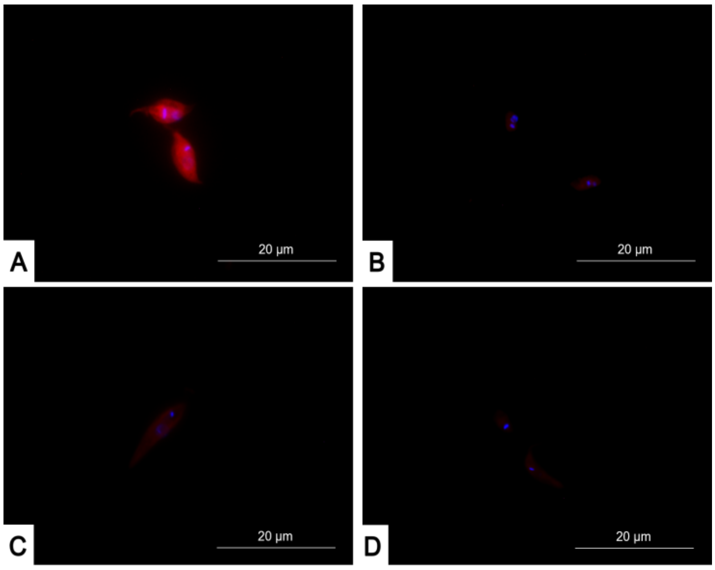

Figure S10. *L. amazonensis* promastigotes incubated with IC_{90} of gongolarone B (B), 6*Z*-1'-methoxyamentadione (C) and 1'-methoxyamentadione (D) during 24 h. Negative control of the parasite without any treatment (A). The 100X images were captured using an EVOS™ FL Cell Imaging System M5000 (Life Technologies, EE. UU.) at λexc = 540 nm and λem = 570 nm. Scale-bar: 20 µm.

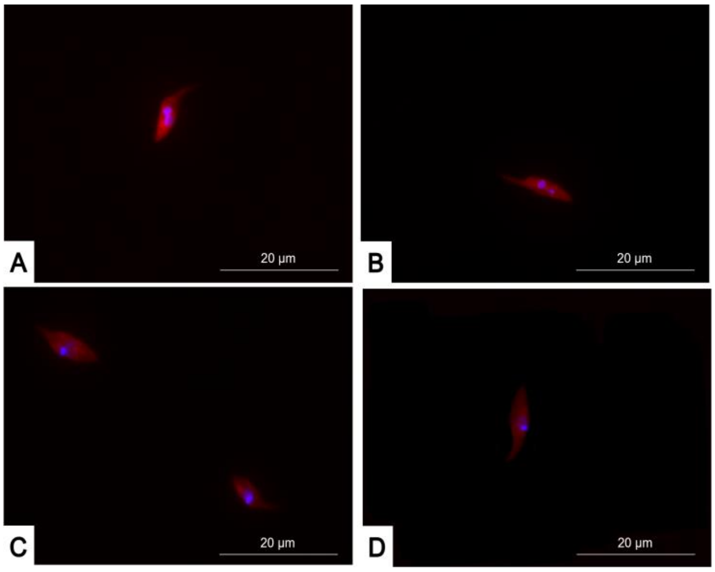

Figure S11. *T. cruzi* epimastigotes incubated with IC_{90} of gongolarone B (B), 6*Z*-1'-methoxyamentadione (C) and 1'-methoxyamentadione (D) during 24 h. Negative control of the parasite without any treatment (A). The 100X images were captured using an EVOS™ FL Cell Imaging System M5000 (Life Technologies, EE. UU.) at λ_{exc} = 540 nm and λ_{em} = 570 nm. Scale-bar: 20 µm.

Immunofluorescence analysis of tubulin

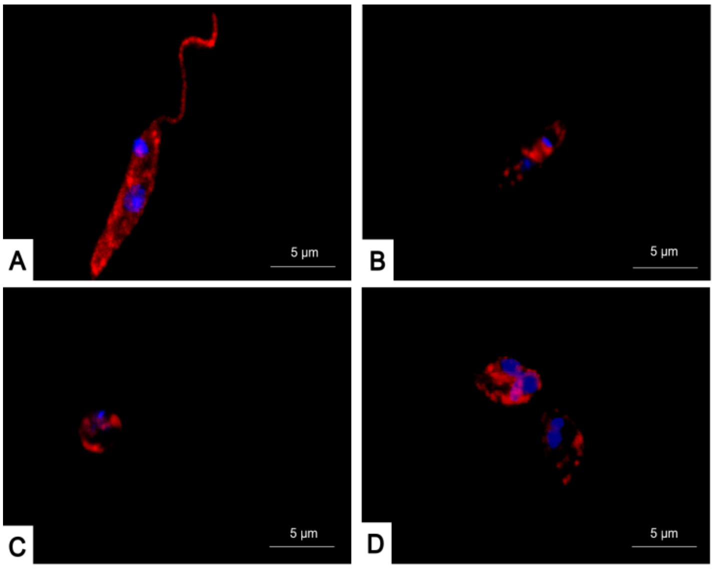

Figure S12. *L. amazonensis* promastigotes incubated with IC_{90} of gongolarone B (B), 6*Z*-1'-methoxyamentadione (C) and 1'-methoxyamentadione (D) during 24 h. Negative control of the parasite without any treatment (A). The images were captured using an inverted confocal microscope Leica DMI 4000 B with a 63x objective (Leica Microsystems, Germany). Scale-bar: 5 µm.

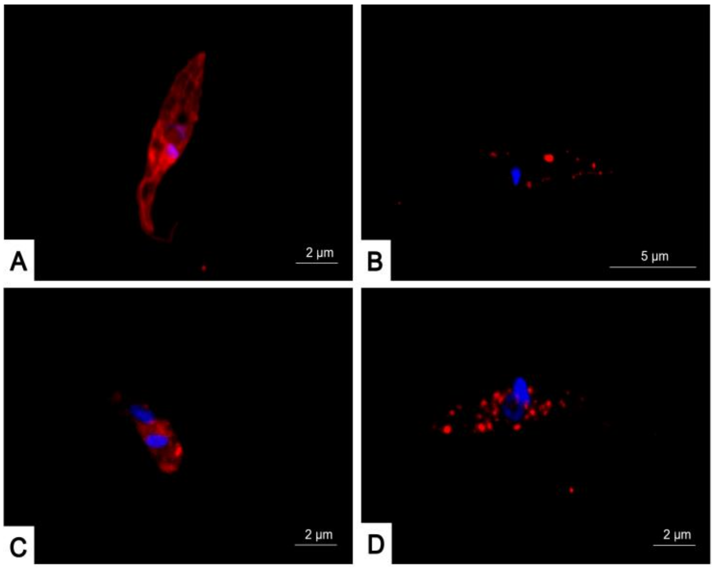

Figure S13. *T. cruzi* epimastigotes incubated with IC_{90} of gongolarone B (B), 6*Z*-1'-methoxyamentadione (C) and 1'-methoxyamentadione (D) during 24 h. Negative control of the parasite without any treatment (A). The 100X images were captured using an inverted confocal microscope Leica DMI 4000 B with a 63x objective (Leica Microsystems, Germany). Scale-bar: 2 and 5 µm.
